# Vaccine strain affects seroconversion after influenza vaccination in COPD patients and healthy older people

**DOI:** 10.1038/s41541-021-00422-4

**Published:** 2022-01-24

**Authors:** Natale Snape, Gary P. Anderson, Louis B. Irving, Andrew G. Jarnicki, Aeron C. Hurt, Tina Collins, Yang Xi, John W. Upham

**Affiliations:** 1 Faculty of Medicine, The University of Queensland Diamantina Institute, Translational Research Institute, Woolloongabba, QLD Australia; 2 Lung Health Research Centre, Department of Biochemistry and Pharmacology, The University of Melbourne, Parkville, VIC Australia; 3 Department of Respiratory Medicine, The Royal Melbourne Hospital, Parkville, VIC Australia; 4 WHO Collaborating Centre for Reference and Research on Influenza, Victorian Infectious Diseases Reference Laboratory, Peter Doherty Institute for Infection and Immunity, The University of Melbourne, Melbourne, VIC Australia; 5 Metro South Health, Princess Alexandra Hospital, Woolloongabba, QLD Australia; 6 Present Address: Department of Infectious Diseases, Roche Pharma Research & Early Development, Basel, Switzerland

**Keywords:** Influenza virus, Chronic obstructive pulmonary disease

## Abstract

Though clinical guidelines recommend influenza vaccination for chronic obstructive pulmonary disease (COPD) patients and other high-risk populations, it is unclear whether current vaccination strategies induce optimal antibody responses. This study aimed to identify key variables associated with strain-specific antibody responses in COPD patients and healthy older people. 76 COPD and 72 healthy participants were recruited from two Australian centres and inoculated with influenza vaccine. Serum strain-specific antibody titres were measured pre- and post-inoculation. Seroconversion rate was the primary endpoint. Antibody responses varied between vaccine strains. The highest rates of seroconversion were seen with novel strains (36–55%), with lesser responses to strains included in the vaccine in more than one consecutive year (27–33%). Vaccine responses were similar in COPD patients and healthy participants. Vaccine strain, hypertension and latitude were independent predictors of seroconversion. Our findings reassure that influenza vaccination is equally immunogenic in COPD patients and healthy older people; however, there is room for improvement. There may be a need to personalise the yearly influenza vaccine, including consideration of pre-existing antibody titres, in order to target gaps in individual antibody repertoires and improve protection.

## Introduction

Chronic obstructive pulmonary disease (COPD) is a common, serious lung disease caused by smoking and exposure to air pollutants^1^. COPD is the third leading cause of mortality worldwide^2^ with the global disease burden likely to increase substantially in coming years^3,4^. Common respiratory viruses such as rhinoviruses and influenza often trigger COPD exacerbations^5,6^, and can lead to secondary bacterial infections, hospitalisations and death^7^. Clinical guidelines recommend influenza vaccination as a priority for COPD patients and other high-risk populations including the elderly and immune compromised^8^. However, vaccine efficacy may be sub-optimal in these high-risk populations, either because of age-related immune dysfunction known as immunosenescence^9–12^, or because of disease-specific deficits in anti-viral immunity^13,14^.

Vaccines act by inducing antibody production and long-lived memory B cells, however, influenza vaccine efficacy can be less than ideal^15^. Post-vaccination, influenza antibody titres decline relatively quickly, particularly in the elderly^16,17^, so annual vaccination is required. Additionally, the influenza virus haemagglutinin (HA) and neuramidase surface proteins exhibit a high propensity for antigenic drift and evasion of host antibodies^18^, so vaccine formulations require updating annually. Strain selection for the vaccine each year is usually based on knowledge of strains circulating in the opposite hemisphere’s winter^19^.

Recent systematic reviews have concluded that influenza vaccination is probably beneficial in COPD, though evidence gaps remain^5^, with relatively few randomised controlled trials (RCTs) directly assessing whether influenza vaccination reduces COPD exacerbations^20^. The largest RCT in the last 50 years was conducted in predominantly vaccine naïve participants. Though this study showed that influenza vaccination reduced COPD exacerbations relative to placebo, it is notable that the active intervention group received double the recommended vaccine dose^21^. In contrast, we previously reported that the humoral immune response to influenza vaccination may be sub-optimal in COPD^13^. There is considerable lack of knowledge regarding the immune response to influenza vaccine in COPD patients—whether the current vaccine strategy is sufficiently immunogenic and whether subgroups of patients fail to mount a robust antibody response. Addressing these knowledge gaps is necessary for developing better vaccine strategies for COPD patients and other at-risk populations.

The aim of this study was to examine vaccine immunogenicity in COPD patients and age-matched healthy older people, in order to identify key variables associated with strain-specific antibody responses. The primary study endpoint was *seroconversion*, defined as ≥four-fold increase in haemagglutination inhibition (HI) antibody titre at 28 days post-inoculation (p.i.). *Seroprotection*, (defined by the World Health Organisation (WHO) as an HI antibody titre ≥1:40) was a secondary endpoint. Notably, this study was not designed to assess whether vaccination reduces the incidence of influenza infections or COPD exacerbations.

## Results

Influenza vaccine formulations were based on Australian Government recommendations and differed slightly across the study years 2015-2017 (Table 1). The H1N1_A/CALIFORNIA/07/2009-like and B_PHUKET/3073/2013-like strains were components in the approved vaccine formulation in all three years of this study, enabling greater statistical power in assessing responses to these strains. A quadrivalent vaccine formulation in 2016 and 2017 added a second B strain (Brisbane/60/2008-like) to the previous trivalent vaccine. One strain differed each year, usually an H3N2 or H1N1 strain.

**Table 1:** WHO-recommended southern-hemisphere influenza vaccine formulations for trivalent and quadrivalent vaccines for each vaccine year.

2015	2016	2017
H1N1_A/CALIFORNIA/07/2009-like	H1N1_A/CALIFORNIA/07/2009-like	H1N1_A/CALIFORNIA/07/2009-like
B/PHUKET/3073/2013-like	B/PHUKET/3073/2013-like	B/PHUKET/3073/2013-like
	B/BRISBANE/60/2008-like	B/BRISBANE/60/2008-like
H3N2_A/SWITZERLAND/ 9715293/2013-like	H3N2_A/HONG KONG/4801/2014-like	H1N1_A/MICHIGAN/45/2015-like

### Participant characteristics

Participant demographics are shown in Table 2. The mean age of COPD participants was 3.8 years higher than that of healthy participants (*p* < 0.01), and just under 70% of COPD participants were male, whereas the healthy participants comprised similar numbers of females and males. Compared with healthy participants, COPD patients had significantly greater cumulative smoke exposure (pack years), were more likely to be current smokers, and more likely to report physician-diagnosed comorbidities, such as asthma and bronchiectasis. Over 90% of our study population received an influenza vaccine in at least one of the two years prior to enrolment. Population demographics were analogous across the Brisbane and Melbourne study sites (Supplementary Table 1).

**Table 2:** Demographics and clinical characteristics of the study population: Comparative between COPD and older healthy participants.

Demographic and Clinical Characteristics	Total	COPD	Healthy	*p* value (COPD vs healthy)
*N*	147	75	72	ns
Female—*n* (%)	60 (40.8)	23 (30.6)	37 (51.4)	0.01
Male—*n* (%)	87 (59.2)	52 (69.3)	35 (48.6)	0.01
Brisbane cohort	94	48	46	ns
Melbourne cohort	53	27	26	ns
2016 returns from 2015 (%)	9 (6)	4 (5.3)	5 (6.9)	
2017 returns from 2016 (%)	9 (6)	5 (6.6)	4 (5.5)	
Age (95% CI)				
Mean	66.8 (65.3–68.3)	68.7 (66.7–70.7)	64.9 (62.7–67.1)	<0.01
Median	67 (64–69)	69 (67–71)	63 (60–68)	
Range	50–90	51–90	50–88	
BMI (95% CI)				
Mean	28.1 (27.0–29.2)	28.3 (26.6–30.0)	27.9 (26.6–29.1)	ns
Median	26.7 (26.1–27.7)	26.6 (24.9–28.0)	26.9 (26.1–28.3)	
Range	18.2–52.9	18.2–52.9	19.0–44.4	
Smoking status *n* (%)				
Never	40 (27.2)	5 (6.6)	35 (48.6)	<0.0001
Former	84 (57.1)	49 (65.3)	35 (48.6)	ns
Current	23 (15.6)	21 (28)	2 (2.8)	<0.0001
Pack Years (95% CI)				
Mean	31.3 (25.6–37.1)	51.6 (43.5–59.7)	10.2 (5.6–14.7)	<0.0001
Median	21.0 (14.0–30.0)	44.5 (39.0–53.0)	0 (0–3)	
Range	0–168	0–168	0–76	
Diabetes—*n* (%)	20 (13.6)	12 (16)	8 (11.1)	ns
Heart condition—*n* (%)	35 (23.8)	23 (30.6)	12 (16.7)	ns
Asthma—*n* (%)	23 (15.6)	18 (24)	5 (6.9)	<0.01
Bronchiectasis—*n* (%)	6 (4.1)	6 (8)	0	<0.05
High blood pressue—*n* (%)	57 (38.8)	33 (44)	24 (33.3)	ns
High cholesterol—*n* (%)	52 (35.4)	27 (36)	25 (34.7)	ns
Mean FEV_1_ predicted % (95% CI)	74.0 (68.6–79.5)	48.7 (43.3–52.0)	102.7 (98.4–107)	<0.0001
Mean FEV_1_/FVC % (95% CI)	62.6 (59.1–66)	50.1 (45.4–54.9)	76.0 (73.8–78.3)	<0.0001
Vaccine history *n* (%)				
Never	5 (3.4)	3 (4)	2 (2.8)	ns
Previous 2 years (both)	120 (81.6)	63 (84)	57 (79.2)	ns
Previous year (only)	11 (7.5)	7 (9.3)	4 (5.5)	ns
Year before previous (only)	4 (2.7)	1 (1.3)	3 (4.2)	ns
Ever before (except previous 2 years)	7 (4.8)	1 (1.3)	6 (8.3)	ns

Significance (*p* values) calculated by Welch’s *t* test (means) and Yates’ Chi-square test (proportions).

*ns* not significant.

Nine subjects participated in the study in consecutive years (2015 and 2016), and another nine participated in both 2016 and 2017. No subjects participated in three consecutive years. Because the influenza vaccine formulation varies by year and because clinical characteristics may fluctuate over time, these ‘repeat participants’ were analysed as individual subjects within each participating year.

### Vaccine-induced antibody response vary by strain

**Fig. 1:**
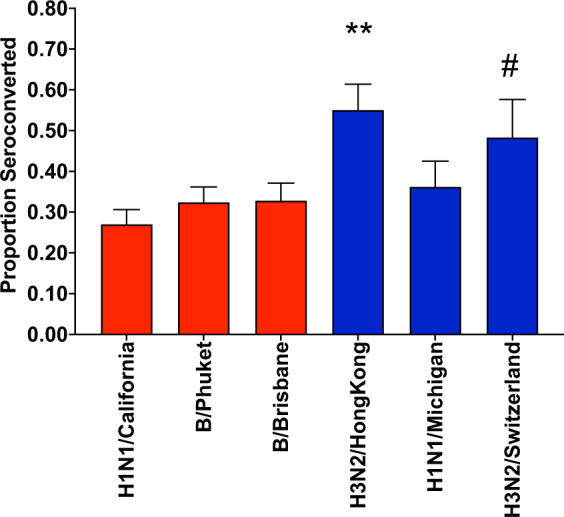
Proportion of subjects that seroconverted (≥ 4-fold rise in Ab titre from D0). Data displayed as proportion ± standard error of fit. Red bars: recurring strains (present in vaccine ≥2 seasons), blue bars: novel strains in vaccine season. **significant difference (*p* < 0.001) to all recurring strains (H1N1/California, B/Phuket & B/Brisbane), ^#^significant difference (*p* < 0.05) to recurring strain H1N1/California only. Significance between proportions calculated by Yates’ Chi-square test.

We compared day 28 p.i. seroconversion rates for those strains included in the vaccine formulation in more than one consecutive year (hereafter referred to as ‘recurring strains’), contrasting this with vaccine strains that were ‘novel’ to a vaccine season (Fig. 1). Notably, a greater proportion of subjects seroconverted to novel strains in a particular year, compared with the recurring strains present in the vaccine formulation every year. For example, the recurring H1N1/California strain and both B vaccine strains of Brisbane and Phuket, elicited seroconversion in 27%, 32% and 31.9% of study participants respectively, whereas the novel strains used in each vaccine season (H3N2/Hong Kong, H1N1/Michigan and H3N2/Switzerland), elicited seroconversion in a larger proportion of study participants: 54%, 36% and 48%, respectively. These differences in seroconversion were statistically significant for H3N2/Hong Kong and H3N2/Switzerland, but not H1N1/Michigan.

**Fig. 2:**
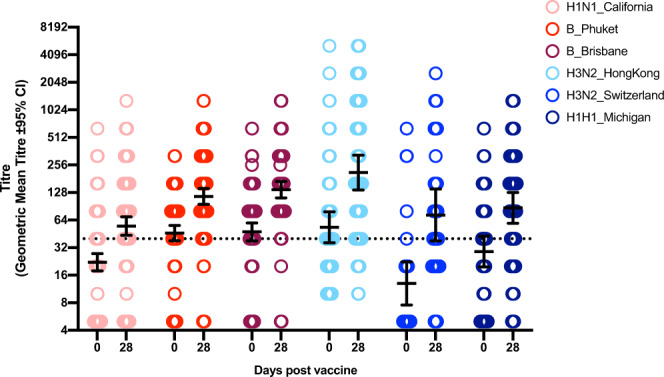
Antibody response pre- (D0) and post- (D28) vaccine for each strain. Data is expressed as GMT ± 95% confidence intervals. Temporal differences for each strain are considered significant if confidence intervals (CI) do not overlap. Dotted horizontal line represents a GMT of 1:40, indicative of seroprotection. GMT: geometric mean titre.

Although the magnitude of post-vaccine antibody response varies considerably between strains, all strains elicited a significantly higher post-vaccine geometric mean titre (GMT) than their corresponding pre-vaccine GMT (Fig. 2). Furthermore, the post-vaccine GMT for all strains increased above 1:40, demonstrating seroprotection. The high pre-vaccine GMT, seen in both B strains and the novel strain H3N2/Hong Kong, indicates that our cohort already exhibited strain-specific seroprotection prior to vaccination. Interestingly, the two novel H3N2 vaccine strains (H3N2/Hong Kong & H3N2/Switzerland) induced greater degrees of post-vaccine seroprotection, with H3N2/Hong Kong also eliciting a higher GMT, than the recurring strains (Fig. 2), signifying that these two novel vaccine strains were particularly efficacious.

### Comparative seroconversion and seroprotection rates

Antibody responses to the various vaccine strains were broadly similar in the COPD and healthy participants. Few differences are observed between COPD and healthy participants in either pre- or post-vaccine seroprotection, or in GMT (Table 3). Vaccine strain B/Phuket elicited a significantly higher seroconversion rate in COPD patients than in healthy participants (adjusted OR *p* = 0.038), and COPD patients had higher pre- and post-vaccine GMT to vaccine strain A/Hong Kong than healthy participants. These differences were not seen with the other strains.

**Table 3:** Vaccine response pre- (D0) and post- (D28) vaccine: Seroprotection rate, Seroconversion rates, and GMT for each vaccine strain.

Vaccine strain	Disease status	*N*	Pre- Seroprotection rate (% of subjects with Ab titres ≥ 1:40)	*p* value	adjusted OR (95% CI)	adj OR *p* value	Post-Seroprotection rate (% of subjects with Ab titres ≥ 1:40)	*p* value	adjusted OR (95% CI)	adj OR *p* value	Seroconverted (% of subjects with 4-fold Ab increase)	*p* value	adjusted OR (95% CI)	adj OR *p* value	Pre-GMT	*p* value	Post-GMT	*p* value
H1N1_A/CALIFORNIA/07/2009-like	Healthy	72	45.8		Ref		76.4		Ref		27.8		Ref		22.9		53.9	
	COPD	75	36	0.58	0.69 (0.34, 1.38)	0.29	70.7	0.55	0.9 (0.4, 1.99)	0.76	26.7	0.97	1.15 (0.53, 2.56)	0.72	20.7	0.56	54.8	0.87
B/PHUKET/3073/2013-like	Healthy	72	63.9		Ref		90.3		Ref		25		Ref		47.1		104	
	COPD	75	74.7	0.8	0.76 (0.31, 1.86)	0.55	92	0.94	1.43 (0.37, 5.55)	0.6	38.7	0.11	2.28 (1.06, 5.09)	0.038*	44.3	0.41	125	0.33
B/BRISBANE/60/2008-like	Healthy	57	72		Ref		91.4		Ref		29.8		Ref		44.4		115	
	COPD	61	80.3	0.39	2.59 (0.88, 7.98)	0.09	96.7	0.38	5.19 (0.91, 43.35)	0.08	34.4	0.74	1.05 (0.43, 2.58)	0.91	52.5	0.820	165	0.14
H3N2_A/SWITZERLAND/9715293/ 2013-like	Healthy	15	6.7		Ref		66.7		Ref		53.3		Ref		9.12		66.5	
	COPD	14	28.6	0.29	10.11 (0.84, 308.50)	0.1	64.3	0.8	1.38 (0.23, 9.09)	0.72	42.8	0.85	0.512 (0.08, 2.82)	0.45	19	0.51	80	0.79
H3N2_A/HONG KONG/4801/2014-like	Healthy	25	48		Ref		92		Ref		56		Ref		29.5		99.9	
	COPD	34	70.6	0.137	2.51 (0.76, 8.69)	0.13	88.2	0.970	0.49 (0.05, 3.41)	0.49	52.9	0.98	0.861 (0.27, 2.66)	0.79	81.6	0.023*	347	0.011*
H1N1_A/MICHIGAN/45/2015-like	Healthy	31	51.6		Ref		77.4		Ref		41.9		Ref		28		87.5	
	COPD	27	51.8	0.81	0.89 (0.30, 2.60)	0.84	74.1	0.99	0.9 (0.26, 3.12)	0.86	29.6	0.48	0.64 (0.20, 1.94)	0.43	30.1	0.95	86.4	0.94

Differences between COPD compared with older healthy subjects calculated by: Wilcoxon ranked sum test (GMT); Chi-Square test with Yates’ correction (proportions); OR calculated from GLM adjusted for gender, age, year, and site.

*Ab* antibody, *GMT* geometric mean titre, *GLM* generalised linear model, *HI* haemagglutination inhibition, *OR* odds ratio; *Ref* reference.

* Significant difference (*p* value ≤ 0.05).

We further assessed whether strain-specific antibody responses varied by study location (Supplementary Table 2). Melbourne participants tended to have higher post-vaccine seroprotection rates and GMT than Brisbane participants, though differences were only significant for the H1N1/California strain (*p* = 0.03, adjOR *p* = 0.029 and GMT *p* ≤ 0.001). The H1N1/California strain also elicited a significantly higher seroconversion rate (*p* = 0.19, adjOR *p* = 0.012) in Melbourne relative to Brisbane participants. Melbourne participants also had higher pre-vaccine seroprotection rates for the P/Phuket (*p* = 0.038) strain and higher pre-vaccine GMT for the H3N2/Hong Kong strain (*p* = 0.031).

Antibody responses were largely similar in women and men (Supplementary Table 3). Pre-vaccination antibody titres to the H1N1/California strain were significantly lower in women than in men, but this difference was not statistically significant after vaccination.

### Regression analyses of antibody responses

Multiple logistic regression analyses indicated that vaccine year, vaccine strain, and study site were all independently associated with the ability to seroconvert at day 28 p.i. (Supplementary Table 4a). Of note, disease status (COPD or healthy) was not independently associated with seroconversion or post-vaccine seroprotection.

Although vaccine strain and year were identified as factors independently associated with seroconversion and seroprotection (Supplementary Table 4a and b, respectively), analysis of the interaction between vaccine strain and year demonstrated that the variation from one year to another could largely be attributed to vaccine strain, with some strains exhibiting collinearity with year (Supplementary Table 9).

Unadjusted univariate linear models suggest that the magnitude of the fold increase in post-vaccine antibody levels was negatively correlated with baseline antibody levels (*p* < 0.0001), and positively correlated with body mass index (BMI; *p* < 0.0001), (Supplementary Table 5). Contrary to expectations, current smoking and total pack years were not associated with seroconversion, whereas cumulative passive smoke exposure was positively associated with ability to seroconvert (Supplementary Table 6).

We also assessed whether any self-reported comorbidities were associated with seroconversion or seroprotection including asthma, bronchiectasis, hypertension, high cholesterol, heart conditions and diabetes. generalised linear model (GLM) analysis indicated that hypertension was the only comorbidity associated with vaccine responses: those with hypertension showed a greater ability to seroconvert (*p* = 0.0491) than those without hypertension (Supplementary Table 7a). However, hypertension was not associated with post-vaccine seroprotection (Supplementary Table 7b).

Participants from Melbourne were more likely to seroconvert than participants from Brisbane (*p* = 0.0178; Supplementary Table 4a) and had a greater likelihood of attaining seroprotection after vaccination (*p* = 0.0130) (Supplementary Table 4b). As noted above, Brisbane and Melbourne study participants had similar demographic and clinical features (Supplementary Table 1).

Baseline neutrophil, monocyte, and eosinophil numbers in whole blood were significantly greater in COPD patients than in healthy participants (Supplementary Fig. 1). However, GLM analysis indicated that these leucocyte populations were not associated with seroconversion or seroprotection at D28 p.i. (Supplementary Table 8a and b, respectively).

## Discussion

We examined immune responses to the seasonal influenza vaccine in COPD patients and healthy elderly people, most of whom had previously been vaccinated. The major finding to emerge was the extent to which vaccine strain was a key independent predictor of seroconversion. The greatest degrees of seroconversion were seen for *novel* vaccine strains, whereas lesser responses were seen with *recurring* vaccine strains. Contrary to our expectations, there was no evidence that COPD patients had sub-optimal vaccine responses relative to healthy older participants.

We have shown that while many participants had high existing antibody titres, indicating they were already protected against certain strains, vaccinating with the same influenza strains in consecutive years failed to significantly augment the antibody response, whereas vaccinating with novel strains was more likely to induce the desired outcome of seroconversion. For example, while study participants showed pre-vaccination GMT at or above the level of protection (HI titre ≥ 40) for both B/ antigens, relatively few individuals seroconverted to these strains. In contrast, the novel H3N2 strains stimulated greater seroconversion rates relative to recurring strains. Similarly, a previous systematic review and meta-analysis has also shown that influenza vaccine effectiveness (VE) can differ greatly by subtype^22^. Tsang et al.^14^ conducted a systems biological approach in healthy adults, in order to develop models that predict responses to influenza perturbation. They discovered that subjects with higher initial titres had lower fold changes at day 70 post-vaccine, also suggesting this inverse correlation may be due to, along with other factors, inhibitory responses in pre-immune subjects^23^. Although older adults in general conventionally respond poorly to influenza vaccines in comparison to younger adults^24^, our study demonstrated that older adults can be more responsive to influenza strains to which they have not been exposed to in preceding years. Others have observed similar trends in adults, whereby participants had relatively high pre-vaccination HI titres but lower rises in post-vaccination HI titres after repeated vaccination, compared with first-time vaccine recipients^25^. Andrew et al. proposed that pre-existing antibodies mask de novo antibody responses, and Huang et al. further suggested that pre-existing antigen-specific antibodies might mask viral epitopes and thereby reduce the magnitude of secondary antibody response to repeated influenza exposure^25,26^. Similarly, Nuñezet al. reported that the impact of pre-existing immunity on responses to influenza vaccination differed between older and younger subjects^27^. It is, however, recognised that older adults are more likely to have been previously exposed to influenza through contact with the virus or vaccination, which can provide a protective effect to influenza via broadly cross-reactive existing cellular immunity^28–30^.

Greater than 90% of our study participants received the influenza vaccine in at least one of the two years prior to our study, and were thus an antigen-experienced population. Participants at this life stage are likely to have previously been exposed to a number of wild-type influenza viral infections, which are known to induce more sustained protection to specific strains than vaccination^31^ and may account for the high pre-vaccine HI levels observed in our study. Evidence from large datasets suggests that repeated vaccination does not impact on the ability of influenza vaccines to reduce hospitalisations due to influenza^32^. A meta-analysis of data from children and adults has also shown no support for a reduction in VE of two consecutive influenza vaccines, however, there is some evidence from this study that serial vaccination from greater than two consecutive seasons may have negative impact on protection^33^. Additionally, high pre-vaccine antibody levels have been shown to reduce the incidence of influenza infections in the elderly^34^.

The COPD cohort in our study included more males, was slightly older, and had more current and prior smoke exposure than the healthy cohort. Despite the demographics not being completely matched between study groups, we observed no significant difference in HI titre, for any strain, between healthy and COPD groups. This is contrary to our previous pilot study, which found lower post-vaccine HI titres to H1N1 antigen in COPD patients compared to healthy participants^35^. The disparity in VE between these two studies may be due the small size of the pilot study, or a mismatch in vaccine antigens in some years, which would reduce ability to mount a suitable immune response in those years^36^. We have also shown herein that disease status (COPD or healthy), current smoking and comorbid disease (aside from hypertension), were not associated with the efficacy of the influenza vaccine, whether assessed by seroconversion or seroprotection. Other studies report similar findings: VE in one older population was not associated with comorbid disease^36^, while another study reported that VE in an elderly, vaccine-naïve population was not associated with COPD severity, age, gender and current smoking status^21^. Given our study population was restricted to a relatively narrow, older range, it is perhaps not surprising that we observed no association between VE and age.

Interestingly, cumulative exposure to passive smoke was positively associated with ability to seroconvert which is unexpected, considering that current smoking and cumulative pack years were not associated with any outcome measures in this study. Further studies in larger cohorts are needed.

Melbourne study participants were more likely to seroconvert than Brisbane study participants, with no demographic or clinical differences between participants from these two large Australian cities. One report suggested that influenza outbreaks are more intense in regions with small population sizes and higher humidity^37^, while others have shown various environmental factors including temperature, humidity and pollution influence the incidence of influenza^38,39^. Given the climatic differences between Brisbane (latitude 27.4° south: sub-tropical) and Melbourne (latitude 37.8° south: temperate), it is possible that differences in key environmental factors such as pollution, sunlight exposure and vitamin D status may impact individual immune response to vaccines^40^. These observations are interesting and warrant further investigations in larger cohorts.

We acknowledge that our study has limitations. The choice of vaccine formulation in each year is regulated by the Australian government, making it difficult to compare specific vaccine strain responses across multiple years. Although we were able to compare longitudinal data for some strains, this was not possible for those strains occurring only in one vaccine season. Accordingly, our study may be slightly underpowered for evaluating these *novel* strains. We considered combining responses from different years for H1 strains and H3 strains, as per McElhaney et al.^41^ and Nunzi et al.^42^, however, we did not use this approach due to the clear variability in reactivity between strains. Circulating strains of influenza vary each year, making it difficult to interpret pre- and post-vaccine HI titres, particularly in regard to cross-reactivity between strains. Furthermore, relying on vaccine-induced antibody response as a correlate of protection against influenza disregards important changes in cellular immunity and enhanced vaccine-mediated protection against influenza in the elderly^11,43^. Data presented in this manuscript are not yet comprehensive enough to allow us to determine the mechanisms involved in such varied response to the chosen annual vaccine strains, yet we speculate that potential mechanisms such as accelerated immunosenescence play a critical role^44^. We continue to look at the underlying mechanisms contributing to poor influenza vaccine responsiveness, and are currently analysing additional data from this study, including strain-specific B cell induction. This is an area of current research in our laboratories. Our study was not powered to evaluate the longer-term benefits of influenza vaccination on COPD exacerbations, though it is important that future studies address this issue. Despite these limitations, our study provides important insights into influenza vaccine responses in healthy older and COPD populations over time, and how these differ for each vaccine antigen.

In light of the recent COVID-19 pandemic, concern has been raised regarding the co-circulation of seasonal influenza and SARS-CoV-2, particularly in vulnerable populations. The overall risk to health and mortality is higher with SARS-CoV-2 than influenza, and it appears that co-infection elicits no worse symptoms than having SARS-CoV-2 alone^45,46^. A study conducted by the national Veterans Health Administration (USA) has, however, indicated the risks for exacerbations of asthma and COPD in this older population to be higher in patients hospitalised with influenza compared with SARS-CoV-2^46^. As the simultaneous circulation of SARS-CoV-2 and influenza strains continues to be a threat to health, it becomes more important for greater uptake of the annual influenza vaccine in these at-risk groups. The capacity to reduce hospitalisations due to COPD exacerbations and other influenza induced indications, alleviating services to better cope with COVID-19 complications, is not insignificant^47,48^.

A central finding of this study is that previous exposure to a specific influenza strain limits the subsequent magnitude of response, or “boosting” ability, to that strain in ensuing seasonal vaccines. Based on seroprotection rates alone, the COPD patients and healthy older participants in our study may appear to be relatively well protected against influenza. However, the findings of Camilloni et al. sound a note of caution in this regard: High rates of infection were seen in an immunised elderly population exposed to mismatched influenza B viruses, even though vaccination had significantly boosted HI titres of cross-reactive antibodies^49^. Although participants in Camilloni et al.’s study were all residing in nursing homes and characteristically frail individuals with potential additional health impacts, concern regarding high rates of viral infection in individuals that still elicit reasonable antibody levels may be extrapolated to immunocompromised individuals such as those with COPD^49^. Furthermore, the rate of decline in antibody titres after vaccination remains troubling, particularly in the elderly where clinical protection is not likely to persist year-round^50^. Recent studies have demonstrated that antibody titres in the elderly are only elevated for 48-56 days after vaccination with the annual, trivalent, split-virus influenza vaccine, consisting of two A strains and one B strain, and may not be further increased by a second booster of this same vaccine^21^.

In conclusion, while our findings provide reassurance that influenza vaccination is immunogenic in both COPD patients and healthy older people, there is clearly room for further improvement. Our findings raise the issue of whether the influenza vaccine should be personalised each year based on pre-existing antibody titres in order to target vaccine formulations to fill gaps in individual antibody repertoires. Moving towards a more individualised seasonal approach, instead of the current blanket recommendation across the population, might increase the efficacy of the influenza vaccine each year, and reduce the burden of influenza in vulnerable groups such as COPD patients. This approach warrants formal testing in well-designed clinical trials.

## Methods

### Study population, ethical and regulatory approvals

This non-randomised, unblinded, observational study was conducted in accordance with the Declaration of Helsinki Principles and the Australian National Health and Medical Research Council (NHMRC) Code of Practice. Ethical approval was granted from local ethics committees: Metro South Health Human Research Committee (approval number: HREC/09/QPAH/297) and The University of Queensland Human Ethics Research Office (approval number: 2011000502). All participants provided written informed consent prior to enrolment.

**Fig. 3:**
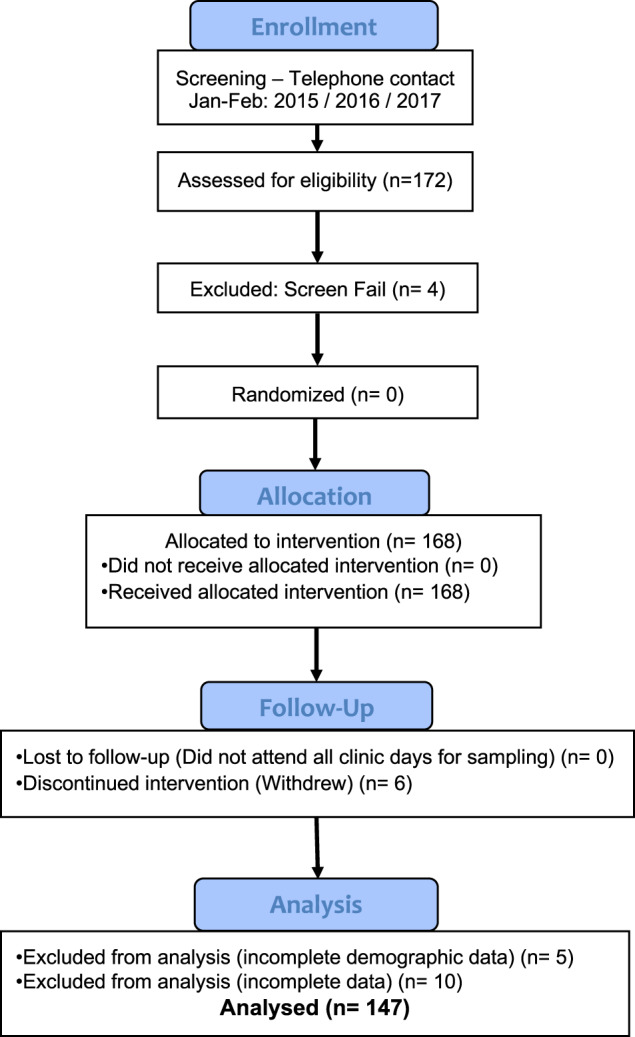
Consort diagram for the study “Using influenza vaccination to understand and improve immune responses to vaccination in patients with COPD and healthy older people (IVC)”. A flow diagram of the progress through the phases of a non-randomised, unblinded, observational study.

Eligible participants aged at or greater than 50 years were recruited from hospitals in two large Australians cities (Brisbane and Melbourne) between 2015 and 2017. COPD patients had a current clinical diagnosis of mild-to-very-severe COPD, a post-bronchodilator forced expiratory volume in one second (FEV_1)_ of <80% predicted, and an FEV_1_/FVC (forced vital capacity) ratio <0.7, with no COPD exacerbations in the 28 days prior to enrolment, and stable medication use. Healthy participants were spouses or partners of COPD patients or were recruited through advertising. A standardised clinical questionnaire was used for screening and assessment. Inclusion and exclusion criteria are further detailed in the Supplementary Methods. Patients reporting additional physician-diagnosed pulmonary diseases were eligible for inclusion provided COPD was the principal pulmonary diagnosis. The consort diagram shows the numbers recruited, screened and enroled in the study (Fig. 3).

### Study design

Recruitment occurred between February and May each study year, prior to the southern-hemisphere winter. Clinical assessment questionnaire, blood collection and spirometry were performed at the first clinic visit (day 0), prior to intramuscular administration of a single standard dose of the seasonal inactivated, trivalent or quadrivalent, split-virion influenza vaccine (FluQuadri™, Sanofi Pasteur). The vaccine consisted of 15 μg HA of each strain without adjuvant: Table 1 lists the vaccine composition in each year. Study participants returned for further blood collection 28 days p.i. to determine serum antibody levels. Further information regarding study design can be found in the Supplementary Methods.

### Immunogenicity

Haemagglutination inhibition (HI) assays were performed against components of each vaccine strain, following pre-treatment of sera with receptor-destroying enzyme, as per methods described by the World Health Organization^51^, and outlined in the Supplementary Methods. *Seroconversion* was defined as fourfold increase in antibody HI titre above 1:40 post-influenza vaccination, also known to be associated with a reduced risk of influenza infection^52^. *Seroprotection* was defined as a HI titre ≥1:40, an antibody level traditionally correlated with reduced risk of influenza infection^53,54^. Blood collection and sample processing are described in the Supplementary Methods. Baseline whole blood leucocyte counts were measured as standard of care.

### Statistical analysis

Descriptive statistics were calculated separately for each vaccine strain. Vaccine responses were described as seroconversion and seroprotection rates and as back-transformed GMT. Participants who did not supply blood samples at both baseline and 28 days p.i. were excluded from the analysis. A *p* value of <0.05 was considered to be statistically significant. Clinical correlations, GLMs and regression models were generated using R (version 4.0.2, 2020, The R Foundation for Statistical Computing Platform, Vienna, Austria). Graphs were generated in R and GraphPad Prism, version 8.4.2 (464), (GraphPad Software, San Diego, CA, USA, www.graphpad.com).

### Clinical study registration

This study is registered with Australian New Zealand Clinical Trials Registry (ANZCTR), under the title “Using influenza vaccination to understand and improve immune responses to vaccination in patients with COPD and healthy older people.” Registration number: ACTRN: ACTRN12620000830998.

### Reporting summary

Further information on research design is available in the Nature Research Reporting Summary linked to this article.

## Supplementary information

Reporting Summary

Supplementary Information

## Data Availability

The data generated as a result of this research project will be managed according to The University of Queensland’s Research Data Management Policy. This policy was developed to ensure that research data are properly managed according to recommendations made in The Australian Code for the Responsible Conduct of Research and applicable legislation. Managed dataset/s associated with this project metadata will also be available to view following a request to the authors (mediated access). This data will be retained for at least five years.
