# Supplementary material for: Vaccine strain affects seroconversion after influenza vaccination in COPD patients and healthy older people
Source: NPJ Vaccines. 2022 Jan 24;7:8. doi: 10.1038/s41541-021-00422-4 (PMC8786852; doi:10.1038/s41541-021-00422-4)
Supplement: Supplementary file 1 — Reporting Summary [file 41541_2021_422_MOESM1_ESM.pdf]

nature portfolio

Corresponding author(s): John Upham, Natale Snape

Last updated by author(s): Aug 27, 2021

Reporting Summary

Nature Portfolio wishes to improve the reproducibility of the work that we publish. This form provides structure for consistency and transparency in reporting. For further information on Nature Portfolio policies, see our Editorial Policies and the Editorial Policy Checklist.

Statistics

For all statistical analyses, confirm that the following items are present in the figure legend, table legend, main text, or Methods section.

n/a	Confirmed	
☐	☒	The exact sample size (n) for each experimental group/condition, given as a discrete number and unit of measurement
☐	☒	A statement on whether measurements were taken from distinct samples or whether the same sample was measured repeatedly
☐	☒	The statistical test(s) used AND whether they are one- or two-sided<br>*Only common tests should be described solely by name; describe more complex techniques in the Methods section.*
☐	☒	A description of all covariates tested
☐	☒	A description of any assumptions or corrections, such as tests of normality and adjustment for multiple comparisons
☐	☒	A full description of the statistical parameters including central tendency (e.g. means) or other basic estimates (e.g. regression coefficient) AND variation (e.g. standard deviation) or associated estimates of uncertainty (e.g. confidence intervals)
☒	☐	For null hypothesis testing, the test statistic (e.g. F, t, r) with confidence intervals, effect sizes, degrees of freedom and P value noted<br>*Give P values as exact values whenever suitable.*
☒	☐	For Bayesian analysis, information on the choice of priors and Markov chain Monte Carlo settings
☒	☐	For hierarchical and complex designs, identification of the appropriate level for tests and full reporting of outcomes
☒	☐	Estimates of effect sizes (e.g. Cohen's d, Pearson's r), indicating how they were calculated

Our web collection on statistics for biologists contains articles on many of the points above.

Software and code

Policy information about availability of computer code

Data collection	No software was used for data collection
Data analysis	R version 4.0.2, 2020, the R Foundation for Statistical Computing Platfor, Vienna, Austria. GraphPad Prism version 8.4.2 (464) (GraphPad Software, San Diego, California USA.

For manuscripts utilizing custom algorithms or software that are central to the research but not yet described in published literature, software must be made available to editors and reviewers. We strongly encourage code deposition in a community repository (e.g. GitHub). See the Nature Portfolio guidelines for submitting code & software for further information.

Data

Policy information about availability of data

All manuscripts must include a data availability statement. This statement should provide the following information, where applicable:

- Accession codes, unique identifiers, or web links for publicly available datasets
- A description of any restrictions on data availability
- For clinical datasets or third party data, please ensure that the statement adheres to our policy

The data generated as a result of this research project will be managed according to The University of Queensland's Research Data Management Policy. This policy was developed to ensure that research data is properly managed according to recommendations made in The Australian Code for the Responsible Conduct of Research and applicable legislation. Managed dataset/s associated with this project metadata will also be available to view either by a DOI listed in the record (open access), or following a request to the authors (mediated access). This data will be retained for at least five years

Field-specific reporting

Please select the one below that is the best fit for your research. If you are not sure, read the appropriate sections before making your selection.

☒ Life sciences ☐ Behavioural & social sciences ☐ Ecological, evolutionary & environmental sciences

For a reference copy of the document with all sections, see nature.com/documents/nr-reporting-summary-flat.pdf

Life sciences study design

All studies must disclose on these points even when the disclosure is negative.

Sample size	Based on pilot data, a sample size of 36 COPD patients and 36 control subjects in each age strata (56-64 years, 65-74 years, and over 75 years) has a power of 90% to detect a difference between patients and controls in post-vaccination antibody titre at a significance level of 0.05. Thus a total of 108 COPD and 108 Control subjects will need to complete the study. On the assumption that 20% of participants will fail to attend one or more of the scheduled blood collections, we estimate that 135 COPD patients and 135 control subjects will need to be recruited. A final total of 168 participants were able to be recruited within the study period.
Data exclusions	Data was excluded if participant did not attend all clinic days, discontinued intervention (withdrew from study), had incomplete results from study analysis or incomplete demographic data.
Replication	Assays were conducted in duplicate replications.
Randomization	Participants were not randomised. Covariates were controlled for via multivariate regression.
Blinding	Blinding was not relevant to this study. The influenza vaccine is given as standard of care to all individuals in this age group and was not considered and intervention in this study.

Reporting for specific materials, systems and methods

We require information from authors about some types of materials, experimental systems and methods used in many studies. Here, indicate whether each material, system or method listed is relevant to your study. If you are not sure if a list item applies to your research, read the appropriate section before selecting a response.

Materials & experimental systems

n/a	Involved in the study	
☒	☐	Antibodies
☒	☐	Eukaryotic cell lines
☒	☐	Palaeontology and archaeology
☒	☐	Animals and other organisms
☐	☒	Human research participants
☐	☒	Clinical data
☒	☐	Dual use research of concern

Methods

n/a	Involved in the study	
☒	☐	ChIP-seq
☒	☐	Flow cytometry
☒	☐	MRI-based neuroimaging

Human research participants

Policy information about studies involving human research participants

Population characteristics	1. Population characteristics recorded included: Age and gender.<br>2. Clinical assessment, including history of respiratory disease and other co-morbidities, history of previous influenza vaccination within the preceding 2 years.<br>3. Measurement of body mass index<br>4. Frailty Assessment Form (COPD participants only and healthy participants age 70 years or greater)<br>5. Spirometry (Existing spirometry data is valid if within 3 months of Day 0 visit)<br>6. COPD severity will be determined with the 'DOSE index', a multi-dimensional staging system that includes dyspnoea, airflow obstruction (FEV1), smoking status and exacerbations per year<br>7. Baseline blood sample 50.5mL (Including FBC if not done within 1 month of Day 0 visit)
Recruitment	Recruitment occurred between February and May each study year, prior to the southern hemisphere winter. Patients were recruited primarily from Respiratory outpatient clinics, supplemented if necessary by patients and controls recruited by advertisements in local newspapers. Control subjects will be spouses or partners of patients. Eligible participants aged 50 years or above were recruited from hospitals in two large Australians cities (Brisbane and Melbourne) between 2015 and 2017. All participants provided written informed consent prior to enrolment.

Ethics oversight	Metro South Health Human Research Committee (HREC/09/QPAH/297) and The University of Queensland Human Ethics Research Office (2011000502) approved the study.

Note that full information on the approval of the study protocol must also be provided in the manuscript.

Clinical data

Policy information about clinical studies

All manuscripts should comply with the ICMJE guidelines for publication of clinical research and a completed CONSORT checklist must be included with all submissions.

Clinical trial registration	ACTRN: ACTRN12620000830998
Study protocol	Study protocol is available on Australian New Zealand Clinical Trials Registry (ANZCTR), under the title "Using influenza vaccination to understand and improve immune responses to vaccination in patients with chronic obstructive pulmonary disease (COPD) and healthy older people." https://www.anzctr.org.au/ACTRN12620000830998.aspx
Data collection	Data was collected between February and May each study year, prior to the southern hemisphere winter, from hospitals in two large Australians cities (Brisbane and Melbourne) between 2015 and 2017. Patients were recruited primarily from established Royal Melbourne Hospital COPD cohort and from the outpatient clinics of the Princess Alexandra Hospital and the Mater Misericordiae Hospital, Brisbane, and Royal Melbourne Hospital, Melbourne. Data was collected at days 0, 1, 7 (optional) 28 and 90 (optional).
Outcomes	Primary outcome: Proportion of influenza vaccine recipients achieving either 'seroprotection' (defined as haemagglutination inhibition antibody titre ≥1:40) or 'seroconversion'<br>Secondary outcome: Identify biomarkers that predict sub-optimal antibody response to influenza vaccine.
