# Supplementary material for: Vaccine strain affects seroconversion after influenza vaccination in COPD patients and healthy older people
Source: NPJ Vaccines. 2022 Jan 24;7:8. doi: 10.1038/s41541-021-00422-4 (PMC8786852; doi:10.1038/s41541-021-00422-4)
Supplement: Supplementary file 2 — Supplementary Information [file 41541_2021_422_MOESM2_ESM.pdf]

Vaccine strain affects seroconversion after influenza vaccination in COPD patients and healthy older people

Supplementary Methods

Study population

Eligible participants 50 years of age or over, were recruited through the respiratory outpatients' clinic at Princess Alexandra Hospital and Mater Hospital (Brisbane), and the established Royal Melbourne Hospital COPD cohort between 2015 and 2017. Patients were eligible for enrolment if co-morbidities were stable and well controlled, and use of inhaled or continuous low-dose oral corticosteroid was stable in the 28 days preceding vaccination and remained stable for the study duration. Study exclusions included; invasive malignancy within the past two years, renal impairment (eGFR < 40 mL/min), acute febrile illness with fever >38.5°C, hypersensitivity to egg proteins, use of oral prednisolone or equivalent ≥10 mg/day, or use of other immunosuppressive therapy. 76 COPD patients of median age 69 years (range: 66 - 71) and 72 healthy participants, median age 63 years (range: 60 - 68) were analysed in this study.

Demographic, clinical assessment and spirometry were recorded at the initial clinic visit. Blood samples taken at baseline (day 0) and 28 days p.i. (± 3 days), were collected in either lithium-heparin tubes or serum tubes. Seasonal approved vaccine was administered to each participant by a clinical research nurse, at day 0 clinic visit.

Immunogenicity

HI assays were conducted by the WHO Collaborating Centre for Reference and Research on Influenza, Victorian Infectious Diseases Reference Laboratory, Peter Doherty Institute for Infection and Immunity, The University of Melbourne, Victoria, Australia. Briefly, sera were serially diluted two-fold in PBS (1:10 to 1:1280), incubated with 4 haemagglutinating units of vaccine strain-specific influenza antigen, and 1% turkey erythrocytes for H1N1 and B strains, or 1% guinea pig erythrocytes in the presence of oseltamivir for H3N2 strains. Titres below the assay's limit of detection (<10 or

<20 respectively) were arbitrarily designated a value half the threshold of detection. Titres were read as the reciprocal of the highest dilution that completely inhibited haemagglutination.

Bloods were collected at each sample day in either: lithium-heparin tubes for isolation of peripheral blood mononuclear cells (PBMCs), plasma and granulocytes, using ficoll-paque™ density gradient centrifugation; or serum tubes, for serum separation. Blood samples were processed on the day of collection, and blood components stored at -80°C or in gaseous phase liquid nitrogen, as required.

Statistical analysis

Chi Square tests with Yates' correction assessed differences in sero-conversion and sero-protection between groups. Differences in GMT between strains were determined with Wilcoxon matched-pairs signed-rank tests, and considered significant if confidence intervals (CI) did not overlap. Odds ratios (OR) were adjusted for gender, age, year and sampling site. Binomial logistic regression models were generated to identify variables associated with either sero-conversion or sero-protection. Separate models were used for each combination of these two outcomes and for each vaccine strain. Stepwise regression modelling was performed to assess the weight of variables associated with these responses.

Supplementary Results

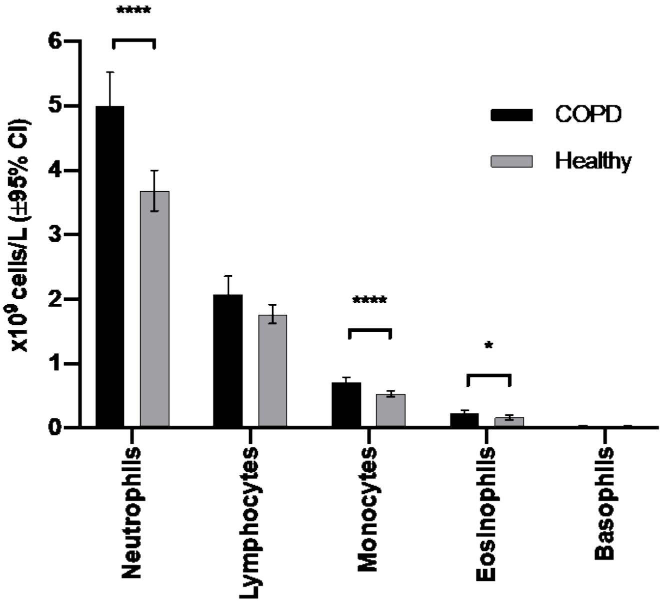

Supplementary Figure 1. Comparison of baseline whole blood cell populations between COPD and healthy participants collected over three seasonal vaccination years 2015/2016/2017. Displayed as number of cells x 10^9 per litre of whole blood ± 95% confidence intervals (** p≤0.0001, * p≤0.05, Wilcoxon ranked sum analysis).**

Supplementary Table 1. Demographic and clinical data: Comparative between participants at both sample sites, Brisbane and Melbourne.

Demographic and Clinical Characteristics	Total	Brisbane	Melbourne	*p*-value
**N**	**147**	**94**	**53**	
**Female - n (%)**	60 (40.8)	38 (40.4)	22 (41.5)	0.963
**Male - n (%)**	87 (59.2)	56 (59.6)	31 (58.5)	0.963
**Healthy**	72 (48.6)	46 (49)	26 (49)	0.875
**COPD**	75 (51)	48 (51)	27 (51)	0.875
**2016 Returns from 2015 (%)**	9 (6.1)			
**2017 Returns from 2016 (%)**	9 (6.1)			
**AGE (95%CI)**				
Mean	66.8 (65.3-68.3)	65.9 (64.1-67.7)	68.49 (65.8-71.2)	0.114
Median	67 (64-69)	65 (63-69)	69 (63-74)	
Range	50-90	50-87	53-90	
**BMI (95%CI)**				
Mean	28.1 (27.0-29.2)	28.8 (27.3-30.2)	27 (25.4-28.5)	0.174
Median	26.7 (26.1-27.7)	27.2 (26.1-29.4)	26.6 (24.7-27.5)	
Range	18.2 - 52.9	18.2-53	18.9-46	
**Smoking status n (%)**				
Never	40 (27.2)	25 (26.6)	15 (28.3)	0.976
Former	84 (57.1)	53 (56.4)	31 (58.5)	0.940
Current	23 (15.6)	16 (17)	7 (13.2)	0.707
**Pack Years (95%CI)**				
Mean	31.3 (25.6-37.1)	30 (22.8-37.1)	33.8 (23.8-43.9)	0.574
Median	21 (14.0-30.0)	21.5 (9.5-31)	18 (9-41)	
Range	0 - 168	0-168	0-120	
**Diabetes - n (%)**	20 (13.6)	14 (14.9)	6 (11.3)	0.721
**Heart condition - n (%)**	35 (23.8)	18 (19.1)	17 (32)	0.117
**Asthma - n (%)**	23 (15.6)	13 (13.8)	10 (18.9)	0.568
**Bronchiectasis - n (%)**	6 (4.1)	3 (3.2)	3 (5.7)	0.770
**High blood pressue - n (%)**	57 (38.8)	36 (38.3)	21 (39.6)	0.985
**High cholesterol - n (%)**	52 (35.4)	30 (31.9)	22 (41.5)	0.323
**Mean FEV_1 predicted % (95%CI)**	74 (68.6-79.5)	70.1 (64-76.3)	80.9 (70.4-91.5)	0.104
**Mean FEV_1/FVC % (95%CI)**	62.6 (59.1-66)	64.6 (60.5-68.7)	58.9 (52.7-65.1)	0.098
**Vaccine History n (%)**				
Never	5 (3.4)	3 (3.2)	2 (3.8)	0.774
Previous 2 years (both)	120 (81.6)	78 (83)	42 (79.2)	0.734
Previous year (only)	11 (7.5)	6 (6.4)	5 (9.4)	0.727
Year before previous (only)	4 (2.7)	2 (2.1)	2 (3.8)	0.951
Ever before (except previous 2 years)	7 (4.8)	5 (5.3)	2 (3.8)	0.985

Supplementary Table 2. Vaccine response pre- (D0) and post- (D28) vaccine comparison between sites (Brisbane and Melbourne). Sero-protection rate: proportion of subjects with HI titre ≥ 1:40. Sero-conversion rate: proportion of subjects eliciting a ≥ 4-fold rise in Ab HI titre from baseline to D28 p.i. Differences in GMT calculated by Wilcoxon ranked sum test. Differences in proportions calculated by Chi Square test with Yates' correction. OR calculated from GLM adjusted for gender, age year and disease status. *p*-values of ≤ 0.05 is considered significant. Ab: antibody; GMT: geometric mean titre; GLM: generalised linear model; HI: haemagglutination inhibition; OR: odds ratio; Ref: reference.

**Vaccine Strain**	**Stie**	**No. Subjects**	**Pre- Seroprotection rate (percentage of subjects with Ab titres ≥1:40)**				**Post- Seroprotection rate (percentage of subjects with Ab titres ≥1:40)**				**Seroconverted (percentage of subjects with 4-fold Ab increase)**				**Pre- GMT**		**Post- GMT**	
			% Positive	*p* -value	adjusted OR (95% CI)	adj OR *p* -value	% Positiv	*p* - value	adjusted OR (95% CI)	adj OR *p* -value	% Positiv	*p* - value	adjusted OR (95% CI)	adj OR *p* -value	GMT	*p*- value	GMT	*p* - value
**H1N1_A/CALIFORNIA/07/ 2009-like**	Bris	94	39.4		Ref		67		Ref		20.2		Ref		19.7		40.9	
	Melb	53	49	0.333	1.56 (0.75, 3.24)	0.231	84.9	**0.03***	2.78 (1.15, 7.32)	**0.029***	39.6	**0.019***	2.78 (1.26, 6.3)	**0.012***	26	0.223	90	**<0.001***
**B/PHUKET/3073/2013-like**	Bris	94	70.2		Ref		87.2		Ref		29.8		Ref		39.4		97.6	
	Melb	53	86.8	**0.038***	1.66 (0.63, 4.72)	0.320	98.1	0.054	4.65 (0.75, 90)	0.162	35.8	0.567	1.66 (0.76, 3.65)	0.204	59.2	0.064	150	0.059
**B/BRISBANE/60/2008-like**	Bris	65	76.9		Ref		93.8		Ref		33.8		Ref		44.8		130.2	
	Melb	53	75.5	0.973	1.29 (0.45, 3.85)	0.630	94.3	0.780	1.79 (0.32, 12.2)	0.517	30.2	0.386	0.72 (0.29, 1.72)	0.46	53.3	0.561	146	0.370
**H3N2_A/SWITZERLAND/97 15293/2013-like**	Bris	29	17.2				65.5				48.3				13		72.7	
	Melb	0	–	–	–	–	–	–	–	–	–	–	–	–	–	–	–	–
**H3N2_A/HONG KONG/4801/2014-like**	Bris	30	50		Ref		86.7		Ref		60		Ref		34.8		136.1	
	Melb	29	72.4	0.134	2.72 (0.85, 9.43)	0.1	93.1	0.699	2.4 (0.39, 20.35)	0.365	28.6	0.520	0.61 (0.20, 1.81)	0.38	81.9	**0.031***	312.4	0.057
**H1N1_A/MICHIGAN/45/20 15-like**	Bris	34	55.9		Ref		73.5		Ref		29.4		Ref		29.5		69.4	
	Melb	24	45.8	0.626	0.69 (0.23, 2.06)	0.510	79.2	0.855	1.22 (0.34, 4.68)	0.759	45.8	0.315	2.06 (0.67, 6.52)	0.206	28.3	0.834	120	0.168

Supplementary Table 3. Pre- and Post-vaccine response (D28 p.i.) comparing female and male subjects. Sero-protection rate: proportion of subjects with HI titre ≥ 1:40. Sero-conversion rate: proportion of subjects eliciting a ≥ 4-fold rise in Ab HI titre from baseline to D28 p.i. Differences in GMT calculated by Wilcoxon ranked sum test. Differences in proportions calculated by Chi Square test with Yates' correction. OR calculated from GLM adjusted for site, age year and disease status. *p*-values of ≤ 0.05 is considered significant. Ab: antibody; GMT: geometric mean titre; GLM: generalised linear model; HI: haemagglutination inhibition; OR: odds ratio; Ref: reference.

**Vaccine Strain**	**Disease status**	**No. Subject**	**Pre- Seroprotection rate (percentage of subjects with Ab titres ≥1:40)**				**Post- Seroprotection rate (percentage of subjects with Ab titres ≥1:40)**				**Seroconverted (percentage of subjects with 4-fold Ab increase)**				**Pre- GMT**		**Post- GMT**	
			% Positive	*p*-value	adjusted OR (95% CI)	adj OR *p*-value	% Positive	*p*-value	adjusted OR (95% CI)	adj OR *p*-value	% Positiv	*p*-value	adjusted OR (95% CI)	adj OR *p*-	GMT	*p*-value	GMT	*p*-value
**H1N1_A/CALIFORNIA/07/2009-like**	Female	60	33.3		Ref		76.6		Ref		31.7		Ref		16.8		47.6	
	Male	87	49.4	0.077	2.16 (1.07, 4.46)	**0.033***	71.3	0.590	0.772 (0.34, 1.71)	0.528	24.1	0.415	0.671 (0.31, 1.46)	0.312	26	**0.032***	59.6	0.19
**B/PHUKET/3073/2013-like**	Female	60	75		Ref		93.3		Ref		38.3		Ref		41.4		116	
	Male	87	77	0.932	1.22 (0.51, 2.94)	0.65	89.7	0.634	0.498 (0.11, 1.84)	0.316	27.6	0.232	0.477 (0.22, 1.01)	0.056	48.8	0.624	113	0.938
**B/BRISBANE/60/2008-like**	Female	47	72.3		Ref		95.7		Ref		29.8		Ref		41.8		125	
	Male	71	78.9	0.551	1.99 (0.67, 6.12)	0.217	92.9	0.818	0.442 (0.05, 2.54)	0.389	33.8	0.798	0.976 (0.39, 2.43)	0.958	53.4	0.598	146	0.279
**H3N2_A/SWITZERLAND/9715293/2013-like**	Female	13	23.1		Ref		69.2		Ref		46.1		Ref		15.3		68.2	
	Male	16	12.5	0.798	1.45 (0.50, 4.28)	0.495	62.5	0.989	0.528 (0.14, 1.81)	0.321	50	0.867	1.45 (0.30, 7.69)	0.644	11.4	0.603	76.6	1
**H3N2_A/HONG KONG/4801/2014-like**	Female	20	50		Ref		85		Ref		55		Ref		36.1		135	
	Male	39	66.6	0.337	1.35 (0.38, 4.58)	0.634	92.3	0.671	2.77 (0.4, 19.8)	0.29	53.8	0.848	1.06 (0.33, 3.47)	0.918	64.6	0.189	254	0.155
**H1N1_A/MICHIGAN/45/2015-like**	Female	27	48.1		Ref		81.5		Ref		37		Ref		27.2		84.2	
	Male	31	54.8	0.806	1.35 (0.46, 4.07)	0.587	70.9	0.531	[illegible].548 (0.14, 1.92	0.358	35.5	0.88	0.897 (0.29, 2.72)	0.847	30.6	0.727	89.5	0.747

Supplementary Table 4: Binomial GLM determining which variables influence Ab response:

a) **Dependent variable = antibody sero-conversion (Fold change of ≥ 4) at day 28 p.i. Year, Strain, Site and Age are significant variables effecting ability to attain a 4-fold increase in Ab titre.**

<table>
<tr><td colspan="5">glmfold <- glm(fold2 ~ Disease2 + sex2 + YEAR + Strain + site2 + AGE + BMI data = data, family = "binomial")</td></tr>
<tr><td>summary(glmfold)</td><td>Estimate</td><td>Std. Error</td><td>z value</td><td>Pr(>|z|)</td></tr>
<tr><td>Intercept</td><td>825.3109</td><td>331.9323</td><td>2.4860</td><td>0.0129*</td></tr>
<tr><td>Disease2healthy</td><td>-0.1751</td><td>0.1966</td><td>-1.8910</td><td>0.3730</td></tr>
<tr><td>sex2m</td><td>-0.2026</td><td>0.1936</td><td>-1.0460</td><td>0.2954</td></tr>
<tr><td>YEAR</td><td>-0.4096</td><td>0.1646</td><td>-2.4880</td><td>0.0128*</td></tr>
<tr><td>StrainA_HK</td><td>1.0549</td><td>0.3322</td><td>3.1750</td><td>0.0015**</td></tr>
<tr><td>StrainA_MICH</td><td>0.7234</td><td>0.3638</td><td>1.9880</td><td>0.0468*</td></tr>
<tr><td>StrainA_SWZ</td><td>0.5693</td><td>0.4580</td><td>1.2430</td><td>0.2139</td></tr>
<tr><td>StrainB_BRIS</td><td>0.2660</td><td>0.2837</td><td>0.9380</td><td>0.3485</td></tr>
<tr><td>StrainB_PHU</td><td>0.2053</td><td>0.2619</td><td>0.7840</td><td>0.4332</td></tr>
<tr><td>site2Melb</td><td>0.4802</td><td>0.2026</td><td>2.3700</td><td>0.0178*</td></tr>
<tr><td>AGE</td><td>-0.0158</td><td>0.0108</td><td>-1.4670</td><td>0.1425</td></tr>
<tr><td>BMI</td><td>0.0185</td><td>0.0145</td><td>1.2750</td><td>0.2022</td></tr>
</table>

b) **Dependent variable = antibody sero-protection (Ab titre of ≥1:40) at day 28 p.i.**

<table>
<tr><td colspan="5">glmsero <- glm(t2sero ~ Disease2 + sex2 + YEAR + Strain + site2 + AGE + BMI data = data, family = "binomial")</td></tr>
<tr><td>summary(glmfold)</td><td>Estimate</td><td>Std. Error</td><td>z value</td><td>Pr(>|z|)</td></tr>
<tr><td>Intercept</td><td>-1218.0000</td><td>425.3000</td><td>-2.8630</td><td>0.0042*</td></tr>
<tr><td>Disease2healthy</td><td>-0.1108</td><td>0.2637</td><td>-0.4200</td><td>0.6743</td></tr>
<tr><td>sex2m</td><td>-0.3249</td><td>0.2654</td><td>-1.2240</td><td>0.2209</td></tr>
<tr><td>YEAR</td><td>0.6052</td><td>0.2109</td><td>2.8690</td><td>0.0041*</td></tr>
<tr><td>StrainA_HK</td><td>1.0430</td><td>0.4809</td><td>2.1680</td><td>0.0302*</td></tr>
<tr><td>StrainA_MICH</td><td>-0.4923</td><td>0.4212</td><td>-1.1690</td><td>0.2425</td></tr>
<tr><td>StrainA_SWZ</td><td>0.4694</td><td>0.4919</td><td>0.9540</td><td>0.3399</td></tr>
<tr><td>StrainB_BRIS</td><td>1.4250</td><td>0.4467</td><td>3.1910</td><td>0.0014**</td></tr>
<tr><td>StrainB_PHU</td><td>1.3270</td><td>0.3590</td><td>3.6960</td><td>0.0002***</td></tr>
<tr><td>site2Melb</td><td>0.7547</td><td>0.3039</td><td>2.4840</td><td>0.0130*</td></tr>
<tr><td>AGE</td><td>-0.0208</td><td>0.0144</td><td>-1.4490</td><td>0.1475</td></tr>
<tr><td>BMI</td><td>0.0094</td><td>0.0195</td><td>0.4850</td><td>0.6274</td></tr>
</table>

Supplementary Table 5: Univariate analysis using magnitude of Ab fold increase at day 28 p.i. as dependent variable

lm <- lm(Fold ~ log(ABT0), data = data)				
	**Estimate**	**Std. Error**	**t value**	**Pr(>|t|)**
Intercept	24.4020	2.5931	9.4100	<2e-16 ***
log(ABT0)	-4.6174	0.6828	-6.7630	3.43e-11***

lm <- lm(Fold ~ BMI, data = data)				
	**Estimate**	**Std. Error**	**t value**	**Pr(>|t|)**
Intercept	-8.8921	4.4026	-2.0200	0.0439*
BMI	0.6059	0.1531	3.9570	0.0001***

Supplementary Table 6: Binomial GLM determining which smoking exposure variables are associated with sero-conversion (Fold change of ≥ 4) at day 28 p.i.

	Estimate	Std. Error	z value	Pr(>|z|)
Intercept	-0.9875	0.1588	-6.2180	5.04e-10***
packyr	0.0020	0.0029	0.7010	0.4831
passivyr	0.0114	0.0045	2.5640	0.0103*
currently smoking	0.0518	0.2859	0.1810	0.8561

Supplementary Table 7: Binomial GLM looking at comorbid diseases as variables influencing Ab response:

a) Dependent variable = antibody sero-conversion (fold increase ≥4) at day 28 p.i.

```
glmmorb1 <- glm(fold2 ~ asthma+bronch+hiblood+hichol+heartcond+diabetes+AGE, data = data, family = "binomial")
```

	Estimate	Std. Error	z value	Pr(>|z|)
Intercept	0.0345	0.6976	0.0490	0.9606
Asthmatic	-0.4084	0.2621	-1.5580	0.1192
Bronchiectasis	0.3648	0.4521	0.8070	0.4198
High blood pressure	0.4018	0.2042	1.9670	0.0491*
High cholesterol	-0.2234	0.2147	-1.0410	0.2981
Heart condition	-0.2997	0.2338	-1.2820	0.1999
Diabetes	0.5164	0.2742	1.8830	0.0597
Age	-0.0107	0.0107	-1.0060	0.3145

b) Dependent variable = antibody sero-protection (Ab titre of ≥1:40) at day 28 p.i.

```
glmmorb1 <- glm(t2sero ~  asthma  + bronch +hiblood + hichol + heartcond  + diabetes +AGE, data = data, family = "binomial")
```

	Estimate	Std. Error	z value	Pr(>|z|)
Intercept	2.6958	0.8961	3.0080	0.00263**
Asthmatic	0.2990	0.3513	0.8510	0.3947
Bronchiectasis	-0.0433	0.5748	-0.0750	0.9400
High blood pressure	0.0264	0.2628	0.1000	0.9200
High cholesterol	-0.0448	0.2721	-0.1640	0.8693
Heart condition	0.3020	0.3013	1.0020	0.3162
Diabetes	-0.1610	0.3590	-0.4490	0.6537
Age	-0.0166	0.0135	-1.2330	0.2175

Supplementary Table 8: Binomial GLM determining which whole blood cells are variables influencing Ab response:

a) Dependent variable = antibody sero-conversion (fold increase ≥4) at day 28 p.i.

glm(formula = fold2 ~ Disease2 + neutro + lympho + mono + eosino + baso, family = "binomial", data = data)

	Estimate	Std. Error	z value	Pr(>|z|)
Intercept	-0.4873	0.3349	-1.4550	0.146
Disease, healthy	-0.1788	0.1964	-0.9110	0.362
neutro	-0.0894	0.0584	-1.5310	0.126
lympho	0.1186	0.0955	1.2410	0.215
mono	0.5252	0.4097	1.2820	0.200
eosino	-0.8861	0.5888	-1.5050	0.132
baso	-2.0469	2.7463	-0.7450	0.456

glmcell2 <- glm(t2sero ~ Disease2 + neutro + lympho + mono + eosino + baso, data = data, family = "binomial")

	Estimate	Std. Error	z value	Pr(>|z|)
Intercept	1.2204	0.4138	2.9490	0.0032**
Disease, healthy	0.0316	0.2514	0.1230	0.9001
neutro	-0.0309	0.0691	-0.4480	0.6544
lympho	0.2659	0.1582	1.6810	0.0927
mono	-0.0138	0.5510	-0.0250	0.9801
eosino	-0.2501	0.7148	-0.3500	0.7264
baso	3.4067	3.7241	0.9150	0.3603

Supplementary Table 9: Testing the interaction between Strain and Year. Year alone becomes not significant, indicating all variation in fold2 is likely to be attributed to strain rather than year.

<table>
<tr><td colspan="5">glmfold2 <- glm(fold2 ~ YEAR* Strain, data = data, family = "binomial")</td></tr>
<tr><th></th><th>Estimate</th><th>Std. Error</th><th>z value</th><th>Pr(>|z|)</th></tr>
<tr><td>Intercept</td><td>-487.4354</td><td>511.9693</td><td>-0.952</td><td>0.3410</td></tr>
<tr><td>YEAR</td><td>0.2413</td><td>0.2539</td><td>0.950</td><td>0.3420</td></tr>
<tr><td>StrainA_HK</td><td>1.2105</td><td>0.3277</td><td>3.694</td><td>0.000221***</td></tr>
<tr><td>StrainA_MICH</td><td>0.2330</td><td>0.3794</td><td>0.614</td><td>0.5392</td></tr>
<tr><td>StrainA_SWZ</td><td>1.2129</td><td>0.5253</td><td>2.309</td><td>0.020952*</td></tr>
<tr><td>StrainB_BRIS</td><td>4290.221</td><td>1061.005</td><td>4.044</td><td>5.26e-05***</td></tr>
<tr><td>StrainB_PHU</td><td>1343.084</td><td>701.760</td><td>1.914</td><td>0.0556</td></tr>
<tr><td>YEAR:StrainA_HK</td><td>NA</td><td>NA</td><td>NA</td><td>NA</td></tr>
<tr><td>YEAR:StrainA_MICH</td><td>NA</td><td>NA</td><td>NA</td><td>NA</td></tr>
<tr><td>YEAR:StrainA_SWZ</td><td>NA</td><td>NA</td><td>NA</td><td>NA</td></tr>
<tr><td>YEAR:StrainB_BRIS</td><td>-2.1276</td><td>0.5262</td><td>-4.043</td><td>5.263-05***</td></tr>
<tr><td>YEAR:StrainB_PHU</td><td>-0.6660</td><td>0.3481</td><td>-1.914</td><td>0.0557</td></tr>
</table>
